# An enigmatic case of cortical anopsia: Antemortem diagnosis of a 14-3-3 negative Heidenhain-variant MM1-sCJD

**DOI:** 10.1080/19336896.2019.1706703

**Published:** 2019-12-27

**Authors:** Julius Obergassel, Lisa Lohmann, Sven G. Meuth, Heinz Wiendl, Oliver Grauer, Christopher Nelke

**Affiliations:** Department of Neurology with Institute of Translational Neurology, University Hospital Münster, Münster, Germany

**Keywords:** Creutzfeld-Jakob disease, Heidenhain, MM1, stroke, FDG-PET, RT-QuIC

## Abstract

Sporadic Creutzfeldt-Jakob disease is the predominant type of human prion disease. While routine diagnostic in phenotypic cases has advanced considerably, the clinical heterogeneity and rarity of subtypes continue to constitute a major clinical and diagnostic challenge. Here, we report a peculiar case of the Heidenhain-variant of MM1 sporadic Creutzfeldt-Jakob disease presenting as a stroke mimic in an 81-year-old patient with a rapid and clinically distinct course of disease as compared to previously reported cases. While 14-3-3 protein was negative, clinical findings substantiated by 18^F^-FDG-PET imaging and RT-QuIC-Assay were able to establish the diagnosis. We conclude that in cases presenting with rapid progressive dementia secondary to sudden cortical anopsia the Heidenhain-variant of CJD should be considered.

## Introduction

The rarity, multifaceted clinical presentation and heterogeneity of human prion diseases render them a major diagnostic and clinical challenge. Sporadic forms of Creutzfeldt-Jakob disease (sCJD) represent the largest group within human prion diseases wherein the MM1-subtype with a homozygous methionine at codon 129 of the PrP^Sc^-type 1 prion protein constitutes the predominant molecular subtype. Although the clinical characteristics of CJD are heterogeneous, they typically include rapidly progressive dementia, psychiatric disorders, ataxia, visual symptoms and an akinetic state of mutism [1]. The disease is incurable and leads to death within months [2]. Initial presentation with isolated visual symptoms is characteristic for the Heidenhain-variant of CJD [3], which accounts for nearly 5% of all CJD cases and typically tests positive for the MM1-subtype [4,5].

Here, we report a peculiar case of an 81 years old female patient diagnosed with MM1-sCJD presenting with an acute and isolated loss of visual acuity mimicking a stroke.

## Case presentation

### Admission and history

Initially, an 81-year-old, Caucasian woman presented with acute loss of visual acuity accompanied by a decline in her general condition and frontotemporal cephalgia. The visual symptoms manifested acutely 2 days prior to admission. Before the onset of visual symptoms, the patient was otherwise healthy, routinely practising competitive dancing. Her medical history is unremarkable except for a pre-existing rotating vertigo which was considered Meniere’s disease earlier. The patient’s husband added progressive olfactory dysfunction during the last months and intermittent accidental falls but negated a relevant cognitive decline. The patient´s father died from an unknown rapidly progressive neurological disease at around 80 years.

### Examinations following admission

The initial neurological examination revealed psychomotor retardation, a loss of visual acuity and raised the suspicion of a right homonymous hemianopsia. Ophthalmologic examination confirmed these findings. An explanatory pathology of the eye was not found. Cranial MRI showed left occipitomesial diffusion restriction in the DWI sequences (diffusion-weighted imaging) with a correlate in the ADC-map (apparent diffusion coefficient) matching the right-sided hemianopsia. Auxiliary findings were global brain volume loss and microangiopathic white matter changes. Other diagnostics including blood (including laboratory panels, inflammatory markers and infection serology) and CSF analysis (including cell count, total protein, direct and indirect virology, **Sup. 1**), 24-hour ECG, neurovascular ultrasound and transthoracic echocardiography did not reveal explanatory findings. The patient was admitted to the neurological ward for further diagnostics under the initial working diagnosis of ischaemic stroke in the posterior cerebral artery territory.

### Examinations in the further course of 7 weeks

**Figure 1.**
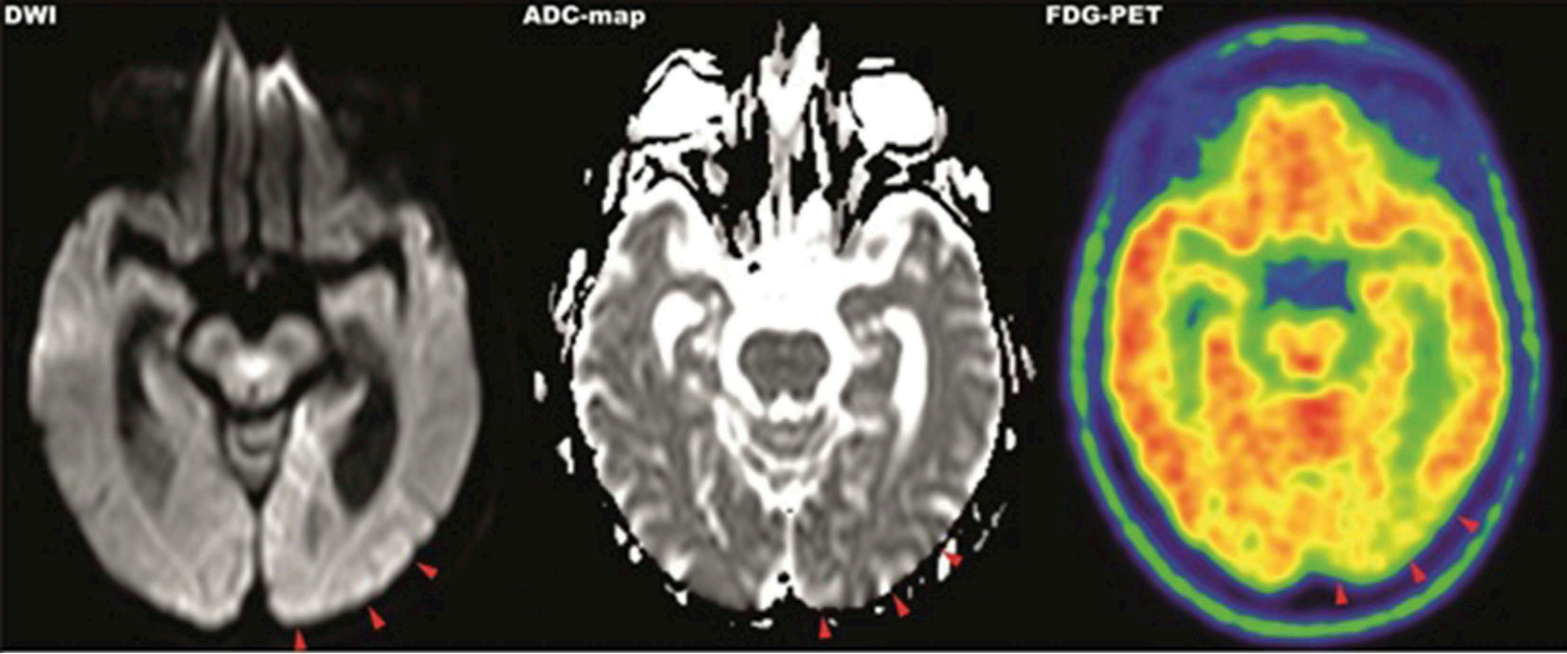
**MRI-DWI (left), ADC-map (middle) and ^18^F-FDG-PET (right) imaging of parietooccipital cortex**. MRI imaging showed hyperintense signals in the left occipitoparietal cortex in diffusion-weighted imaging with correlating hypointense signals in the ADC-map. The same area presents hypometabolic in 18^F^-FDG-PET imaging.

**Figure 2.**
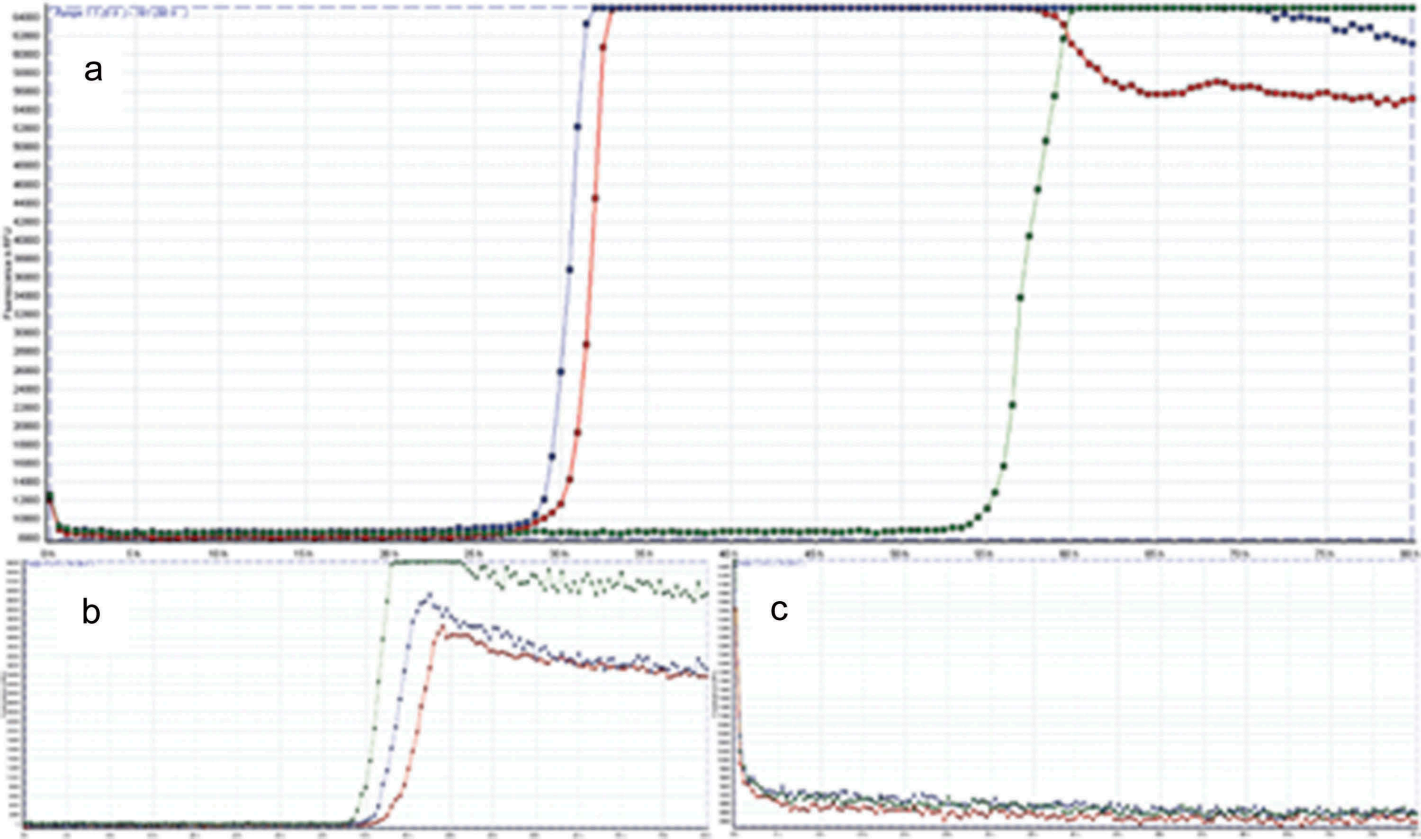
**Result of the RT-QuIC assay**. All graphs display fluorescence intensity in relative fluorescence units (RFU) on vertical over horizontal time axis in hours. RT-QuIC of patient samples is displayed in graph A, graph B shows the curve of the positive control and graph C of the negative control, respectively. An increase of fluorescence during reaction above the cut-off of 10,000 RFU is considered a positive result.

Within the next 7 weeks the patient´s cognitive status including her vigilance declined. She demonstrated intermittent somnolence, dysarthria, visual and acoustic hallucinations, progressive loss of visual acuity, ideomotor apraxia and ataxia. Interim working diagnosis of progressive supranuclear palsy (PSP) was raised, when a supranuclear vertical gaze palsy together with postural instability and bradykinesia developed. However, an L-dopa test did not improve neurological symptoms and advanced ^18^F-FDG-PET/CT-imaging (fluorine-18 fluorodeoxyglucose positron emission tomography/computed tomography) revealed significant hypometabolism in the left occipital lobe and adjacent left parietotemporal and precuneal cortex areas but not in PSP-typical medial frontal cortex areas, providing a functional correlate for the progressive visual dysfunction (Figure 1). Further results from CSF analysis including neuropathological diagnostics, immunofluorescence tests and an encephalitis antibody panel (**Sup. 1**) did not yield aberrant findings. However, an analysis of dementia markers in CSF from a re-puncture 2.5 weeks after admission revealed a massively elevated Tau-protein (>2200 pg/ml) along with normal amyloid levels. As left frontotemporal periodical sharp waves were detected in an electroencephalogram, the new suspect diagnosis of CJD was raised. Phosphorylated neurofilament heavy chain (pNfH) in CSF was strongly elevated (2064 pg/ml). Although further analysis of CSF did not detect 14-3-3 protein, a real-time quaking-induced conversion assay (RT-QuIC) PrP^Sc^-aggregation analysis [6] identified pathological protein aggregations of PrP^Sc^ 1 protein (Figure 2). PRNP gene was amplified from whole-blood-extracted genomic DNA via polymerase-chain-reaction (PCR) and homozygosity for methionine at codon 129 was detected via restriction fragment length polymorphism (RFLP).

In the further course of the disease, the patient gradually developed spastic hypertonia, global bradykinesia and a loss of consciousness cumulating in respiratory insufficiency and ultimately death 7 weeks after hospital admission and 9 weeks after the onset of symptoms. The patient did not consent to an autopsy. Further genetic analysis excluded familial CJD.

## Discussion

Here, we report a noteworthy presentation of the Heidenhain-variant, a phenotype constituting approximately 5% of the total sCJD cases. While visual symptoms are present in up to 20% to 35% of sCJD – more often in younger patients [7] – the diagnostic criteria for the Heidenhain-variant require isolated visual symptoms at the onset of the disease. In the further course of the disease, cognitive, pyramidal, extrapyramidal and cerebellar symptoms usually manifest. The case reported here differs considerably from previously reported cases of the Heidenhain-variant of sCJD. With a symptom onset at 81 years, our patient was approximately 10 to 15 years older than previously described mean ages at disease onset [4,8]. Remarkably, the patient died 9 weeks after the initial onset of symptoms. Thus, the course of disease was strikingly shorter than the average of reported cases of Heidenhain-variant sCJD presenting with 16 to 24 weeks disease duration [2,4,5,8]. Of note, this time course is already shorter compared to other subtypes of sCJD. Accentuating the velocity of disease, the initial acute and isolated loss of visual acuity combined with homonymous hemianopsia were considered an ischaemic stroke on admission.

In respect to the diagnostic work-up, an initial MRI revealed a hyperintense DWI-signal in the correlating cortex area compatible with sCJD, being present in up to 80% of sCJD cases [7]. The concurrent onset of a minor ischaemic stroke in the posterior cerebral artery territory prior to admission cannot be ruled out, however, given the further course of the disease and the initial clinical presentation, appears unlikely. Although a lumbar puncture was performed on admission, the lack of additional neurological symptoms and the rapid onset complicated the diagnosis as standard CSF markers of dementia, such as beta amyloid or tau protein, were not included initially and only analysed 2 weeks after admission. As of yet, the lack of a systematic review precludes a conclusive statement regarding the positivity of CSF 14-3-3 in Heidenhain-variant sCJD. However, a literature search identified 37 cases of Hv-sCJD in which CSF was definitely tested and reported. Out of these cases, 29 (78,4%) were positive, 2 (5,4%) were ambiguous and 6 (16,2%) were negative for CSF 14-3-3 [9–13]. Sensitivity and specificity of 14-3-3 detection vary largely between different studies but sensitivity has been reported to be in a range of 61% to 95% [14,15]. Therefore, negative testing for 14-3-3, in this case, did not rule out sCJD; however, it delayed the diagnosis until confirmation via RT-QuIC prion protein aggregation assay [6]. A possible diagnostic short-coming is the late employment of the EEG. Simple and cost-effective, an earlier EEG might have provided CJD-typical findings at an earlier stage of the disease as it does in approximately 60% of all cases and with a sensitivity of 65% in those cases with a disease duration shorter than 6 months [16]. Topologically concordant abnormalities in MRI imaging and ^18^F-FDG-PET imaging provided a symptom matching correlate with lesions in the parietooccipital cortex. Similar locations of hypometabolism were reported very rarely in other Heidenhain-variant cases when ^18^F-FDG-PET imaging was applied [9,17,18]. Broadly, ^18^F-FDG-PET was suggested to be of diagnostic relevance in the differential diagnosis of sCJD from other forms of rapidly progressive dementia in a few cases and series. The parietooccipital cortex was mainly affected with hypometabolism in patients with visual symptoms which is in line with our findings [19,20]. However, the diagnostic relevance of ^18^F-FDG-PET imaging in differential diagnosis of Heidenhain-variant sCJD remains elusive due to a lack of prospective trials but can be considered in unclear cases of rapidly progressive dementia.

RT-QuIC analysis was developed to allow for a more rapid and specific discriminability between treatable and non-treatable pathologies [6,21,22]. Second generation RT-QuIC (**Sup. 2**) showed sensitivity rates around 95% and a specificity of 100% in retrospective and one prospective study [23–25], with even higher sensitivities up to 100% for genetic variants of CJD [26,27]. The impact of RT-QuIC testing for the diagnosis of CJD is currently discussed by involved medical societies and acknowledged in national guidelines [14,28,29]. Given the specificity and in accordance with the current guidelines from the German Society for Neurology, we consider the positive RT-QuIC assay in conjuncture with the clinical presentation and supporting findings convincing for the diagnosis of sCJD. However, we also acknowledge that RT-QuIC has not yet been established as a routine laboratory tool for the definite diagnosis of CJD.

In conclusion, we established the probable antemortem diagnosis of Heidenhain-variant sCJD with MM1 molecular subtype. Key clinical findings were an advanced age at onset combined with an accelerated course of disease mimicking an ischaemic stroke. We propose that an unusual presentation of the Heidenhain-variant might be considered as a ‘stroke-mimic’ in cases of isolated visual symptoms followed by unexplained neurological deterioration. FDG-PET imaging and CSF RT-QuIC prion protein aggregation assay have proven their diagnostic value, while we were unable to detect 14-3-3 protein. A limitation of our case is the lack of a histopathology. The clinical heterogeneity of sCJD sustains diagnostic difficulties.
