# Supplementary material for: An enigmatic case of cortical anopsia: Antemortem diagnosis of a 14-3-3 negative Heidenhain-variant MM1-sCJD
Source: Prion. 2019 Dec 27;14(1):24–8. doi: 10.1080/19336896.2019.1706703 (PMC6959312; doi:10.1080/19336896.2019.1706703)
Supplement: Supplemental Material [file kprn-14-01-1706703-s001.docx]

# Supplement Material 2 – Second generation RT-QuIC method

The real-time quaking-induced conversion (RT-QuIC) assay was developed to make use of PrPSc’s unique feature of converting normal-folded recombinant Prp (rPrPSen) into misfolded PrPSc and therefore amplify an initial small amount of PrPSc and detect the aggregated proteins in a cell-free in-vitro assay [34]. A revised protocol of 2nd generation RT-QuIC with references to previous protocols was recently published by Atarashi et al. as the current standard procedure [35]. Here, CSF, is added to a RT-QuIC reaction mix containing rPrPSen, thioflavin T, ethylenediaminetetraacetic acid tetrasodium salt (EDTA) and a phosphate buffer at pH 7.4 [22]. PrPSc aggregation is detected by thioflavin T fluorescence induced by binding to amyloid at an excitation/emission maximum of 442/482nm) and measured in real-time with a steeper exponential increase in fluorescence which is followed by a slight continuous decrease
