# Supplementary material for: An enigmatic case of cortical anopsia: Antemortem diagnosis of a 14-3-3 negative Heidenhain-variant MM1-sCJD
Source: Prion. 2019 Dec 27;14(1):24–8. doi: 10.1080/19336896.2019.1706703 (PMC6959312; doi:10.1080/19336896.2019.1706703)
Supplement: Supplemental Material [file kprn-14-01-1706703-s002.docx]

# Supplement 1

CSF Analysis

Cell count: Lymphozytes: 0 /µl; Granulozytes: 0 /µl; Erythrozytes: 0 /µl; other cells: 0 /µl; total cell count: 0 /µl (<5) ; Protein: Total protein: 494 mg/l; Albumin CSF: 299.00 mg/l; Albumin Serum: 41.70 g/l; Albumin quotient: 7.2; IgG CSF: 31.80 mg/l; IgG Serum: 9.28 g/l; IgG quotient: 3.4; IgA CSF: 4.47 mg/l; IgA Serum: 3.20 g/l; IgA quotient: 1.4 ; IgM CSF: 0.188 mg/l; IgM Serum: 0.45 g/l; IgM quotient: 0.4;

Oligo clonal IgG: negativ;

Glucose CSF: 68.70 mg/dl (49-75) ; Glucose serum: 106.00 mg/dl (70-130) ; Glucose quotient: 0.65 (0,6-0,9) ; Laktate: 2.02 mmol/l (1,7-2,6) ; Bilirubin: neg.; Hemoglobin: negativ;

Antibody analysis from CSF

IFT (immunofluorescence) on brain tissue: Cerebellum CSF: negativ (negativ) ; Cerebellum serum: negativ (<1:10) ; Hippocampus CSF: negativ (negativ) ; Hippocampus serum: negativ (<1:10) ; Pancreas CSF: negativ; Pancreas serum: negativ (<1:10) ; Colon CSF: negativ; Colon Serum: negativ (<1:10) ;

Auto immune encephalitis IFT on transf. cells: DNER CSF: negativ (negativ) ; DNER serum: negativ (<1:10) ; ZIC4 CSF: negativ (negativ) ; ZIC4 serum: negativ (<1:10) ; NMDA-R (GluN1a) CSF: negativ (negativ) ; NMDA-R (GluN1a) serum: negativ (<1:10) ; CASPR2 CSF: negativ (negativ) ; CASPR2 serum: negativ (<1:10) ; AMPA1/2-R CSF: negativ (negativ) ; AMPA1/2-R serum: negativ (<1:10) ; LG1 CSF: negativ (negativ) ; LG1 serum: negativ (<1:10) ; DPPX CSF: negativ (negativ) ; DPPX serum: negativ (<1:10) ; GABA-B1/2-R CSF: negativ (negativ) ; GABA-B1/2-R serum: negativ (<1:10) ; GAD65 CSF: negativ (negativ) ; GAD65 serum: negativ (<1:10) ;

Auto immune encephalitis immunoblot: Tr(DNER) CSF: negativ (negativ) ; Tr(DNER) serum: negativ (negativ) ; GAD65 CSF: negativ (negativ) ; GAD65 serum: negativ (negativ) ; ZIC4 CSF: negativ (negativ) ; ZIC4 serum: negativ (negativ) ; Titin CSF: negativ (negativ) ; Titin serum: negativ (negativ) ; SOX1 CSF: negativ (negativ) ; SOX1 serum: negativ (negativ) ; Recoverin CSF: negativ (negativ) ; Recoverin serum: negativ (negativ) ; Hu (ANNA-1) CSF: negativ (negativ) ; Hu (ANNA-1) serum: negativ (negativ) ; Yo (PCA-1) CSF: negativ (negativ) ; Yo (PCA-1) serum: negativ (negativ) ; Ri (ANNA-2) CSF: negativ (negativ) ; Ri (ANNA-2) serum: negativ (negativ) ; Ma2/Ta CSF: negativ (negativ) ; Ma2/Ta serum: negativ (negativ) ; CV2 CSF: negativ (negativ) ; CV2 serum: negativ (negativ) ; Amphiphysin CSF: negativ (negativ) ; Amphiphysin serum: negativ (negativ) ;

Total amyloid: 828.00 pg/ml (>500) ; Infection panel: CMV-IgG-AK (ELISA)/S: >250 AU/ml; CMV-IgM-AK (ELISA)/S: negativ; HSV-1 IgG-AK (ELISA)/S: positiv; HSV-1 IgM-AK (ELISA)/S: negativ; HSV-2 IgG-AK (ELISA)/S: negativ; HSV-2 IgM-AK (ELISA)/S: negativ;

Nucleic acid analysis: CMV-PCR (quant.)/L: negativ Cop/ml; EBV-PCR (quant.)/L: negativ Cop/ml; HHV-7-PCR (qual.)/L: negativ; HSV-1 PCR (quant.)/L: negativ Cop/ml; HSV-2 PCR (qual.)/L: negativ; VZV PCR (qual.)/L: negativ; HHV-6 PCR (quant.)/L negativ IE/ml; HHV-8-PCR (qual.)/L negativ;

Serum-CSF-quotient (ASI): HSV-IgG ASI/L: 0.7 Index; VZV-IgG ASI/L: 0.6 Index; CMV-IgG ASI/L: 0.5 Index; Measles-IgG ASI/L: 0.7 Index; Mumps-IgG ASI/L: 0.6 Index; Rubella-IgG ASI/L: 0.6 Index;

14-3-3 negative

Total-tau protein >2200 pg/ml

NSE 15,1 ng/ml

S100b 2,5 ng/ml

PrPSc positiv
